# Resting-state brain network features associated with short-term skill learning ability in humans and the influence of *N*-methyl-d-aspartate receptor antagonism

**DOI:** 10.1162/netn_a_00045

**Published:** 2018-10-01

**Authors:** Zhenxiang Zang, Lena S. Geiger, Urs Braun, Hengyi Cao, Maria Zangl, Axel Schäfer, Carolin Moessnang, Matthias Ruf, Janine Reis, Janina I. Schweiger, Luanna Dixson, Alexander Moscicki, Emanuel Schwarz, Andreas Meyer-Lindenberg, Heike Tost

**Affiliations:** Department of Psychiatry and Psychotherapy, Research Group System Neuroscience in Psychiatry, Central Institute of Mental Health, University of Heidelberg, Medical Faculty Mannheim, Mannheim, Germany; Department of Psychiatry and Psychotherapy, Research Group System Neuroscience in Psychiatry, Central Institute of Mental Health, University of Heidelberg, Medical Faculty Mannheim, Mannheim, Germany; Department of Psychiatry and Psychotherapy, Research Group System Neuroscience in Psychiatry, Central Institute of Mental Health, University of Heidelberg, Medical Faculty Mannheim, Mannheim, Germany; Department of Psychiatry and Psychotherapy, Research Group System Neuroscience in Psychiatry, Central Institute of Mental Health, University of Heidelberg, Medical Faculty Mannheim, Mannheim, Germany; Department of Psychiatry and Psychotherapy, Research Group System Neuroscience in Psychiatry, Central Institute of Mental Health, University of Heidelberg, Medical Faculty Mannheim, Mannheim, Germany; Department of Psychiatry and Psychotherapy, Research Group System Neuroscience in Psychiatry, Central Institute of Mental Health, University of Heidelberg, Medical Faculty Mannheim, Mannheim, Germany; Department of Psychiatry and Psychotherapy, Research Group System Neuroscience in Psychiatry, Central Institute of Mental Health, University of Heidelberg, Medical Faculty Mannheim, Mannheim, Germany; Department of Neuroimaging, Central Institute of Mental Health, University of Heidelberg, Medical Faculty Mannheim, Mannheim, Germany; Department of Neurology and Neurophysiology, Albert-Ludwigs-University, Freiburg, Germany; Department of Psychiatry and Psychotherapy, Research Group System Neuroscience in Psychiatry, Central Institute of Mental Health, University of Heidelberg, Medical Faculty Mannheim, Mannheim, Germany; Department of Psychiatry and Psychotherapy, Research Group System Neuroscience in Psychiatry, Central Institute of Mental Health, University of Heidelberg, Medical Faculty Mannheim, Mannheim, Germany; Department of Psychiatry and Psychotherapy, Research Group System Neuroscience in Psychiatry, Central Institute of Mental Health, University of Heidelberg, Medical Faculty Mannheim, Mannheim, Germany; Department of Psychiatry and Psychotherapy, Research Group System Neuroscience in Psychiatry, Central Institute of Mental Health, University of Heidelberg, Medical Faculty Mannheim, Mannheim, Germany; Department of Psychiatry and Psychotherapy, Research Group System Neuroscience in Psychiatry, Central Institute of Mental Health, University of Heidelberg, Medical Faculty Mannheim, Mannheim, Germany; Department of Psychiatry and Psychotherapy, Research Group System Neuroscience in Psychiatry, Central Institute of Mental Health, University of Heidelberg, Medical Faculty Mannheim, Mannheim, Germany

**Keywords:** Resting-state fMRI, Short-term motor learning, System neuroscience, Functional brain networks, NMDA receptor-related plasticity

## Abstract

Graph theoretical functional magnetic resonance imaging (fMRI) studies have demonstrated that brain networks reorganize significantly during motor skill acquisition, yet the associations between motor learning ability, brain network features, and the underlying biological mechanisms remain unclear. In the current study, we applied a visually guided sequential pinch force learning task and graph theoretical analyses to investigate the associations between short-term motor learning ability and resting-state brain network metrics in 60 healthy subjects. We further probed the test-retest reliability (*n* = 26) and potential effects of the *N*-methyl-d-aspartate (NMDA) antagonist ketamine (*n* = 19) in independent healthy volunteers. Our results show that the improvement of motor performance after short-term training was positively correlated with small-worldness (*p* = 0.032) and global efficiency (*p* = 0.025), whereas negatively correlated with characteristic path length (*p* = 0.014) and transitivity (*p* = 0.025). In addition, using network-based statistics (NBS), we identified a learning ability–associated (*p* = 0.037) and ketamine-susceptible (*p* = 0.027) cerebellar-cortical network with fair to good reliability (intraclass correlation coefficient [ICC] > 0.7) and higher functional connectivity in better learners. Our results provide new evidence for the association of intrinsic brain network features with motor learning and suggest a role of NMDA-related glutamatergic processes in learning-associated subnetworks.

## Author Summary

Learning a new motor skill prompts immediate reconfigurations of distributed brain networks followed by adaptive changes in intrinsic brain circuits related to synaptic plasticity. Here, we identify global brain network properties and a cerebellar-cortical functional subnetwork that are both significantly associated with motor learning ability in a previously trained visuomotor task in humans. We further show that the associated functional subnetwork connectivity but not the global brain network properties are susceptible to ketamine. Our findings suggest a distinct functional role for learning-related global versus local network metrics and support the idea of a preferential susceptibility of learning-associated subnetworks to *N*-methyl-d-aspartate antagonist and plasticity-related consolidation effects.

## INTRODUCTION

The acquisition of new motor skills requires the brain to flexibly reconfigure neural circuits to master a desired performance level (Bassett & Mattar, 2017). Recent studies have demonstrated that different circuits are involved at distinct stages of learning (Dayan & Cohen, 2011; Penhune & Steele, 2012). Whereas the initial learning phase engages a widespread network consisting of primary motor area (M1), supplementary motor area (SMA), basal ganglia (BG), dorsolateral prefrontal cortex (DLPFC), premotor cortex, and posterior cerebellum, the following longer term learning phase relies on a smaller set of brain regions including M1, SMA, BG, and the lateral cerebellum (Dayan & Cohen, 2011). In addition, the specific type of motor learning task determines the preferential involvement of brain regions with sequential learning challenging cortical areas while more complex sensorimotor tasks with novel kinematic additionally challenge the BG and cerebellum (Hardwick, Rottschy, Miall, & Eickhoff, 2013).

The interactions between brain regions during motor learning can be studied in the framework of brain networks. By combining network analysis and functional magnetic resonance imaging (fMRI), recent studies have shown that brain network features including flexibility (Bassett et al., 2011), connectivity strength, local path length, and nodal efficiency (Heitger et al., 2012; Sami & Miall, 2013) change in response to motor learning and can predict its rate (Bassett et al., 2011). Notably, changes in the brain network architecture cannot only be assessed *during* the process of motor learning by using task-based fMRI, but also during rest. Although there is some evidence that intrinsic network connectivity measures derived from prior resting-state fMRI (rs-fMRI) predict motor learning abilities (Mawase, Bar-Haim, & Shmuelof, 2017; Wu, Srinivasan, Kaur, & Cramer, 2014), recent studies also suggest that motor learning effects can be detected using rs-fMRI *after* task practice (Albert, Robertson, & Miall, 2009; Sami & Miall, 2013; Sami, Robertson, & Miall, 2014). However, whereas plasticity-related effects of motor learning likely shape the intrinsic configuration of brain circuits, the biological mechanisms in humans remain largely unknown.

Plausible molecular mechanisms contributing to motor learning–related network changes include glutamate-dependent processes (Dayan & Cohen, 2011). Supportive evidence is provided by animal studies showing that motor training can shift the glutamatergic *N*-methyl-d-aspartate (NMDA) receptor subunit composition in BG (Kent, Deng, & McNeill, 2013) and promote the NMDA-dependent synaptic plasticity in the primary motor cortex of rats (Kida et al., 2016), while impaired motor performance was observed in mGluR4 gene knockout mice (Pekhletski et al., 1996). In humans, evidence for the involvement of glutamate-dependent processes during motor learning is less direct. Here, many studies have focused on the effects of a common functional polymorphism (Val66Met) in the brain-derived neurotrophic factor (BDNF) gene (Fritsch et al., 2010; McHughen et al., 2010), a downstream modulator of the molecular cascade supporting synaptic plasticity linked to motor learning impairments and altered motor cortical activations in the plasticity-impaired Met allele carriers (Fritsch et al., 2010; McHughen et al., 2010; Thomason, Yoo, Glover, & Gotlib, 2009). For the evidence in humans, a study by Hadj Tahar et al. further showed that the NMDA receptor antagonist amantadine significantly impairs motor learning in healthy subjects (Hadj Tahar, Blanchet, & Doyon, 2004).

In the current work we aimed to answer two main questions in healthy humans: first, whether the brain’s resting-state network configuration relates to individual differences in short-term motor learning; and second, whether these metrics can be influenced by NMDA receptor antagonism. We first investigated whether resting-state network features relate to individual differences in short-term motor learning ability by combining an established sequential visual isometric pinch force learning task (Reis et al., 2009) with rs-fMRI and graph theoretical analyses. We hypothesized that both global network diagnostics and functional connectivity among a circumscribed set of brain visuomotor brain areas would relate to individual motor learning ability (Doyon & Benali, 2005; Hikosaka, Nakamura, Sakai, & Nakahara, 2002). Second, we tested whether ketamine influences the functional connectivity of motor learning–related subnetworks. Here, we hypothesized that NMDA receptor blockade would decrease the connectivity of motor learning–related subnetworks.

## MATERIALS AND METHODS

### Participants

Sixty healthy right-handed volunteers (mean age 26.6 ± 7.5 years, 33 men) underwent visuomotor training followed by a resting-state fMRI scan (mean training duration: 26.9 ± 5.7 min; mean time interval between motor training and fMRI scan: 45.8 ± 7.5 min). Exclusion criteria included MRI contraindications, a history of psychiatric and neurological illness, prior head trauma, and current alcohol or drug abuse. None of the subjects had a first-degree relative with a psychiatric disorder or received psychopharmacological treatment. All participants provided written, informed consent for a protocol approved by the Ethics Committee of the University of Heidelberg.

### Visuomotor Learning Task

**Figure 1.**
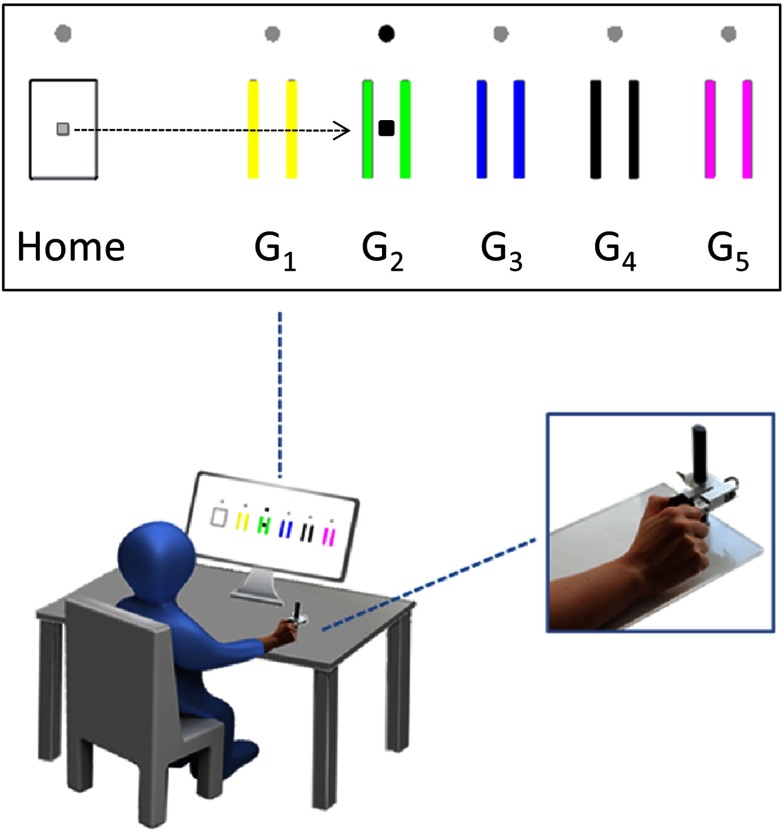
Setup of the sequential visual isometric pinch task (see Materials and Methods for details). Subjects were asked to move the cursor into the highlighted targets (i.e., G_2_) as fast and as accurate as possible. The sequence of targets was 2-5-3-1-4. (Manikin illustration ©Petr Ciz – Fotolia.com).

Behavioral training consisted of a single session with a modified version of an established (Reis et al., 2009) sequential visual isometric pinch force task. Subjects were seated 80 cm in front of a 28-inch monitor depicting a home position and five target gates (G_1_-G_5_, Figure 1) while holding a force transducer between their right thumb and index finger. The application of pinch force moved a screen cursor from the home position in a right hand direction toward the target gates, whereas relaxation resulted in a leftward cursor movement back toward the home position. The distance of the cursor to the home position increased logarithmically with increasing pinch force in order to make the task more difficult. Subjects were instructed to modulate their pinch force so that the cursor navigated as quickly and accurately as possible along the following sequence: *home-G_2_-home-G_5_-home-G_3_-home-G_1_-home-G_4_*. After getting familiar with the setting, subjects performed four training blocks consisting of 35 trials (completed sequences) each. Movement times per trial were measured from movement onset in the home position to stopping at the last gate (*G*_4_). Error rates were calculated as ratio of gates per block with over- or undershooting cursor movements (missed gates).

### Definition of Skill Learning

Following prior work with this task (Reis et al., 2009), we calculated individual skill measures for each block using the following formula:

$$skill\;measure=\ln(\frac{1-error\;rate}{error\;rate(\ln{\left(duration\right)}^{5.424})})$$ (1)

where *duration* is the average movement time across the trials of the block, and *error rate* is the rate of over- and undershoots across the trials of the block (Reis et al., 2009). Over all training blocks, individual differences in skill learning ability were calculated based on the difference in the skill measure between the last and first training block (skill learning = skill measure_block4_ − skill measure_block1_).

### MRI Data Acquisition

Neuroimaging was performed on a 3T MRI scanner (Siemens Trio, Erlangen, Germany) equipped with a 32 channel multi-array head-coil. Details on MRI sequences are given in Supporting Information (Zang, Geiger, Braun, Cao, Zang, Schäfer, … & Tost, 2018).

### fMRI Data Processing

Image processing was performed using standard routines implemented in the Statistical Parametric Mapping software (SPM8, http://www.fil.ion.ucl.ac.uk/spm/software/spm8/) and the Data Processing Assistant for Resting-State fMRI toolbox [DPARSF, (C. Yan & Zang, 2010)]. All images were realigned to the first image of the time series, corrected for slice timing, spatially normalized to the Montreal Neurological Institute (MNI) EPI template, and spatially smoothed with an 8-mm full-width at half-maximum Gaussian kernel. For each participant, we then extracted the mean time series from 264 brain regions derived from the Automated Anatomical Labeling (AAL 116) brain atlas (Tzourio-Mazoyer et al., 2002) by random parcellation (Zalesky, Fornito, Harding, et al., 2010). From the node time series, we regressed out the time series of white matter and cerebrospinal fluid masks (derived from SPM tissue probability maps thresholded at 90% for CSF and 99% for WM; Cao et al., 2014) and the six head motion parameters from the realignment step. The resulting residual time series were temporally filtered using a 0.01–0.1 Hz band-pass filter.

### Quantification of Head Displacements

The functional connectivity estimates and network diagnostics derived from resting-state fMRI may be impacted by motion artifacts (Power, Barnes, Snyder, Schlaggar, & Petersen, 2012; Satterthwaite et al., 2013; C. G. Yan et al., 2013). To account for this, we used in-house software to estimate averaged frame-wise displacement (FD) (Power et al., 2012) and included average FD as covariates of noninterest in our analyses.

### Construction of Connectivity Matrices

For the construction of brain networks, we computed pairwise Pearson correlation coefficients between the processed time series of each node, which resulted in a 264 × 264 two-dimensional matrix for each subject. We then thresholded the matrices in 1% intervals over a range of 40 densities from 1% to 40% to generate binary graphs (e.g., in the 1% thresholded matrix only the top 1% of the highest positive correlations are represented by assigning a value of 1 to the internode connections).

### Calculation and Analysis of Graph Diagnostics

On the global brain network level, graph features were computed using the Brain Connectivity Toolbox (Rubinov & Sporns, 2010). Specifically, for each density, we calculated seven reliable (Cao et al., 2014) global brain network markers that reflect the integration and segregation of whole-brain network and were shown to be in association with cognitive functions (Alavash et al., 2015): Transitivity, characteristic path length, global efficiency, small-worldness, modularity Q (Newman, 2006), assortativity, and mean connectivity coefficient. The detailed descriptions of the seven markers are given in the Supporting Information (Zang et al., 2018).

For association with the degree of skill learning, the network properties were averaged across densities and introduced as a dependent variable into separate linear regression models with skill learning as independent variables of interest and age, sex, and averaged FD as covariates of noninterests. Hochberg’s stepwise *p* value adjustment method (Hochberg, 1988) was used to correct raw *p* values for multiple hypothesis testing.

### Network-Based Statistics

We analyzed the connectivity matrices with network-based statistics (NBS) to identify clusters of node connections associated with skill learning ability. Compared with the mass-univariate testing of independent links, NBS offers higher statistical power by identifying connected components from a set of uncorrected thresholded links that are significantly associated with a variable of interest (Zalesky, Fornito, & Bullmore, 2010), and then uses a randomization approach to evaluate the null hypothesis on the level of connected subclusters (rather than individually for each connection). Following prior procedures (Wang et al., 2013), we defined initial linear regression models for each of the (*N*(*N* − 1))/2 = 34,716 (*N* = 264) possible links in the connectivity matrices. The regression models included skill learning as an independent variable of interest and age, sex, and the averaged FD as covariates of noninterest. From the resulting *p* matrix, we defined a set of suprathreshold connections by isolating all links with *t* > 3.48 and *p* < 5 × 10^−4^ and used *M* = 5,000 permutations (Wang, Zuo, & He, 2010) to estimate the null distribution during permutation testing of the identified cluster association.

### Supplemental Analyses

To further probe the quality of the skill learning–related NBS result, we further (a) quantified the test-retest reliability of the mean connectivity of the identified cerebellar-cortical cluster, (b) considered the potential role of structural confounds by testing the relationship between skill learning and gray matter volume of the nodes contributing to the cluster, and (c) explored the effects of low-dose ketamine as NMDA receptor antagonist on skill learning ability and the connectivity of the identified NBS cluster. Additionally, we aimed to probe (d) the robustness of our results by using a more conservative head motion correction approach and (e) the specificity of the association between motor learning ability and global network features by controlling for the mean functional connectivity as covariate of noninterest. Finally, we examined the identified cerebellar-cortical network association to skill learning with respect to potential effects of the choice of the initial cluster-forming significance threshold and parcellation scheme for NBS, respectively, by exploring the outcome of (f) two additional cluster-forming significance thresholds (*p* < 0.001, *p* < 0.0001) and (g) an alternative whole-brain functional atlas (Rosenberg et al., 2016) containing a comparable number of nodes (268 parcellations) as our AAL-based atlas.

#### Test-retest reliability.

As previous studies have demonstrated that the reliability of functional connectivity estimates is spatially heterogeneous (Mueller et al., 2015), we aimed to establish the robustness of the connectivity estimates in the identified subnetwork before further exploring it in the context of a pharmacological challenge study. To quantify the test-retest reliability of the connectivity phenotype, we reanalyzed the resting-state reliability data reported in Cao et al. (2014). Following the nomenclature of Fleiss (1986), we considered an ICC value below 0.4 as poor, 0.4–0.75 as fair to good, and >0.75 as excellent. Detailed information about fMRI data is given in the Supporting Information (Zang et al., 2018).

#### Structural correlates.

We analyzed the high-resolution structural data with the voxel-based morphometry toolbox (VBM8, http://dbm.neuro.uni-jena.de/vbm8/) by using default parameters. Detailed descriptions of preprocessing the structural data are provided in the Supporting Information (Zang et al., 2018). We then extracted the GM volume of the nodes contributing to the identified NBS cluster and entered the sum GM volume as a dependent variable in a regression model that included skill learning as independent variable of interest and age and sex as covariates of noninterest (significance level: *p* < 0.05).

#### NMDA receptor challenge.

To quantify the effects of the NMDA receptor antagonist ketamine on the identified cerebellar-cortical subnetwork, we analyzed the ketamine challenge data reported in (Francois et al., 2016; Grimm et al., 2015). In this study, resting-state fMRI data were acquired in 24 healthy individuals (12 women, mean age 25 years, mean body weight 70 kg) undergoing three consecutive fMRI sessions over the course of 3 weeks. The pharmacolog ical protocol followed a double blind, placebo-controlled, order randomized, three-period cross-over design with single intravenous doses of either saline (placebo condition), ketamine (0.5 mg/kg body weight), or scopolamine (4 μg/kg body weight). Drug infusions started 73.8 ± 13.8 minutes prior to the resting-state scan and were 40.02 ± 6.02 min in duration. The visuomotor learning task started 15.6 ± 3.5 min after infusion onset and was completed around the end of the infusion (at 40.4 ± 8.61 min). To ensure this, we used the same experimental setup for the training as in the main study except for a slightly shorter training duration (25 trials for each block, 4 blocks in total). Since we did not test a hypothesis assuming effects of mAch-blockade (scopolamine condition) on the identified subnetwork phenotype, we only analyzed the rs-fMRI data from the ketamine and placebo conditions in the current study. For this, we created a covariate of noninterest coding for the order of ketamine and placebo conditions (ketamine first, placebo first) that was included in the applied repeated-measure ANOVA. For pharmacokinetic analysis, blood samples for quantification of norketamine plasma levels were drawn immediately before and after the MRI scan (see Francois et al., 2016, and Grimm et al., 2015, for details). The time interval between ketamine and placebo infusion was 9.6 ± SD 3.5 days. One subject was excluded because of side effects under ketamine (Francois et al., 2016); four more subjects were excluded because they had already participated in either the current (3 subjects) or in other visuomotor learning studies (1 subject). In total, 19 subjects were included in further data analyses. The processing of the behavioral data, rs-fMRI data, node definitions, and construction of connectivity matrices followed the protocol described above. To test for drug effects, we extracted the mean connectivity from the links of the cerebellar-cortical subnetwork identified in the NBS analysis (see Results section) and used a repeated-measures ANOVA with drug as within-subjects factor and age, sex (as factor), body mass index (BMI), the order (as factor) of drug, and the differences of averaged FD (placebo vs. ketamine condition) as covariates of noninterest. To directly relate the connectivity indices of the identified subnetwork to the administration of the drug in the ketamine condition, we quantified intravenous norketamine levels by chromatographic analysis from the blood samples taken immediately prior to the MRI scan. For details on the blood sample processing, please refer to Francois et al. (2016). We used a linear regression model in which the norketamine values were introduced as a dependent variable, the mean connectivity estimates from the network links as independent variable of interest and age, sex, BMI, and averaged FD as nuisance covariates (significance level: *p* < 0.05). Detailed descriptions about drug administration are provided in the Supporting Information (Zang et al., 2018).

#### Controlling for mean individual functional connectivity differences.

As global differences in connectivity strength might directly influence network properties (van den Heuvel et al., 2017), we aimed to replicate our results by using the individual mean functional connectivity average over all connections as an additional covariate of noninterest in our analyses.

#### Scrubbing to correct for head motion.

Since sharp in-scanner motion can introduce systematic, artificial connectivity (Power et al., 2012), we aimed to replicate our findings by using a “scrubbing” approach as described in detail in Power et al. (2012). In short, all frames of the time series with a FD >0.5 mm were removed. Two subjects were excluded from this analysis because their number of spikes exceeded 10% of the total time points, leaving a total of 58 subjects.

## RESULTS

### Skill Learning Ability

**Figure 2.**
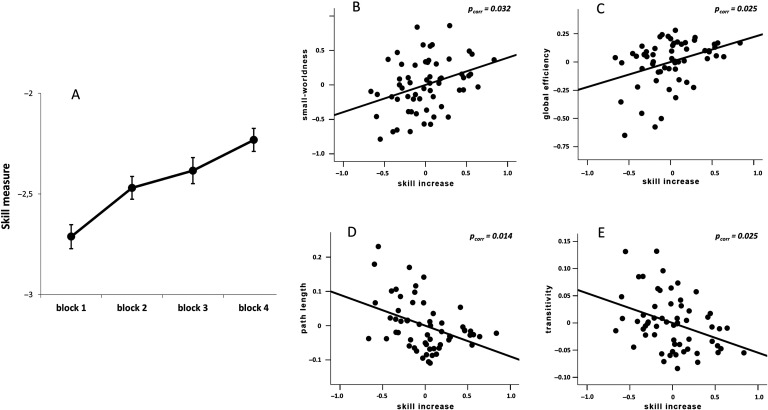
(A) Skill increase in the sequential visual isometric pinch task across the training blocks (dots depict the mean values of the skill measure across blocks). Error bars indicate standard errors. (B–E) Regression plots show significant associations of short-term motor learning ability (block 4 to block 1) and resting-state fMRI-derived graph diagnostics (adjusted for covariates and constant; see Results section for details) after controlling for age, sex, and FD.

Training improved sequential visual isometric pinch task performance as indicated by a significant decrease in the trial durations and error rates and a significant increase in the skill measure (Figure 2A) across blocks (*F*_(3,57)_ values > 4.27, all *p* values < 0.009). The analysis of the skill learning measure confirmed a significant increase in skill performance (skill measure block 4 to block 1) at the end of the training (one-sample *t* test, *t*_(59)_ = 11.43, *p* = 1.2 × 10^−16^).

### Relationship to Graph-Based Diagnostics

At the global brain network level, we observed significant associations between the individual skill learning ability and four of the seven graph diagnostics. Whereas positive correlations were found for small-worldness (*t*_(55)_ = 2.73, *r* = 0.35, *p*_*raw*_ = 0.008, *p*_*corr*_ = 0.032, Figure 2B) and global efficiency (*t*_(55)_ = 2.90, *r* = 0.36, *p*_*raw*_ = 0.005, *p*_*corr*_ = 0.025, Figure 2C), we detected negative associations for characteristic path length (*t*_(55)_ = −3.33, *r* = −0.41, *p*_*raw*_ = 0.002, *p*_*corr*_ = 0.014, Figure 2D) and transitivity (*t*_(55)_ = −2.92, *r* = −0.37, *p*_*raw*_ = 0.005, *p*_*corr*_ = 0.025, Figure 2E). We observed no significant associations between skill learning ability and assortativity and modularity Q of the network (all *p*_*corr*_ values > 0.225; Table S2, Zang et al., 2018). In addition, there was no significant correlation between skill learning ability and whole-brain mean connectivity (*r* = −0.11, *p*_*raw*_ = 0.41; Table S2, Zang et al., 2018). All calculated graphs displayed small-world network properties (σ =γ /λ > 1, range 1.02–2.69).

### Relationship to Subnetwork Functional Connectivity

**Figure 3.**
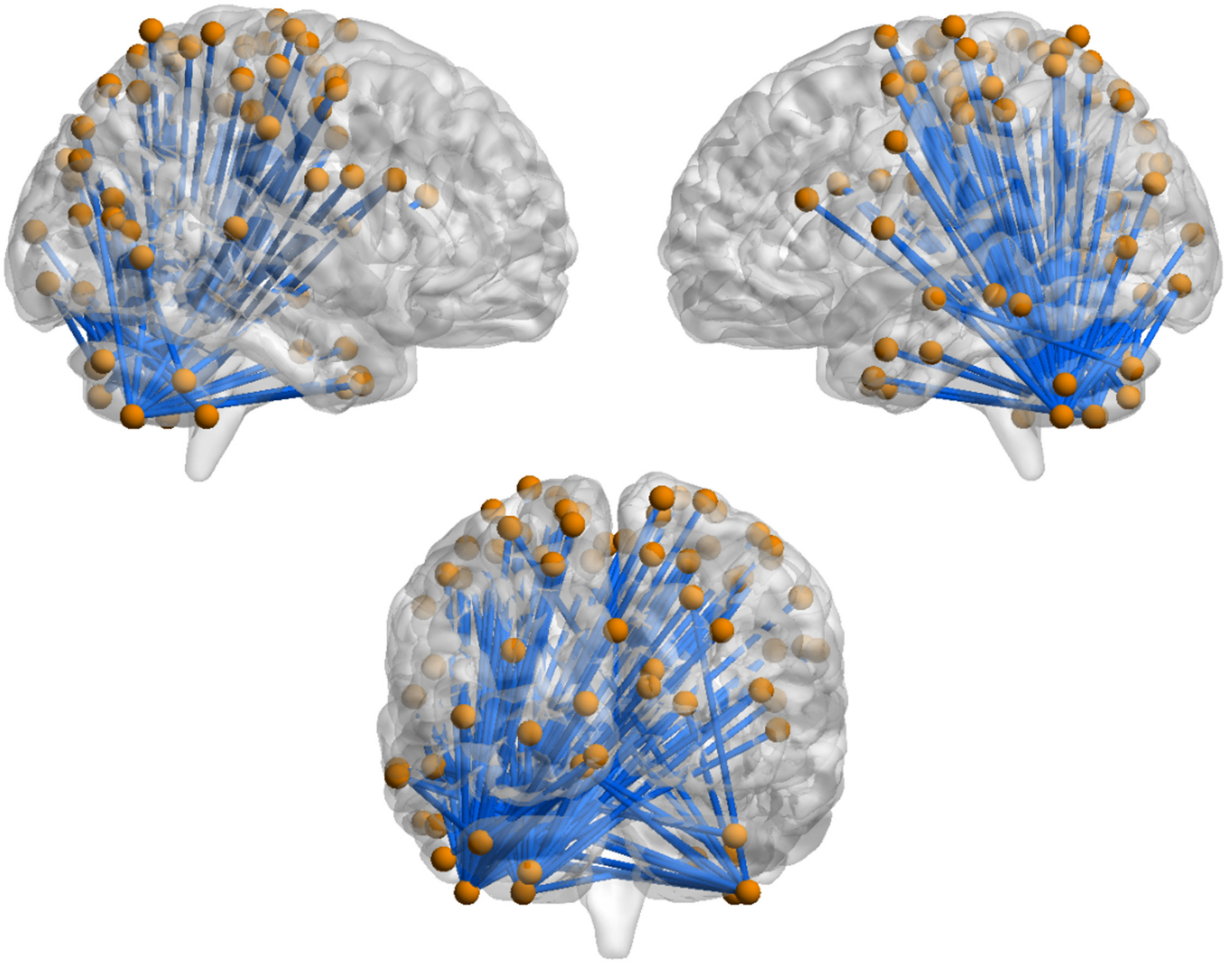
Illustration of the NBS-derived cerebellar-cortical functional network associated with short-term skill learning. Spheres represent center-of-gravity coordinates of the NBS-derived regions. Images are visualized using BrainNet Viewer (Xia, Wang, & He, 2013). Detailed information can be found in Table S1 (Zang et al., 2018).

Although we did not detect a significant association between short-term skill learning ability and the mean correlation estimates of the whole-brain functional connectome (*r* = −0.11, *p*_*raw*_ = 0.41; Table S2, Zang et al., 2018), significantly associated brain subnetworks likely exist. Consistent with this notion, NBS identified a cluster of links with a significant positive association between skill learning ability and the functional connectivity estimates of the cluster links (uncorrected initial *p* < 5 × 10^−4^, FWE corrected *p* = 0.037; Figure 3). The cluster consisted of 69 nodes and 91 links mainly interconnecting the cerebellum, frontal, and parietal lobes. Specifically, most of the links of this cluster connected area 7b and area 8 of the left cerebellum to the cortex, in particular, to nodes mapping in proximity to M1, primary sensory cortex, SMA, dorsal premotor cortex, intraparietal sulcus, and the motion sensitive visual processing area V5. A detailed description of all nodes and links of the identified subnetwork is provided in Table S1 (Zang et al., 2018).

### Supplemental Analyses

#### Test-retest reliability.

The test-retest reliability analysis of the connectivity estimates of the NBS subnetwork yielded an ICC_2,1_ of 0.72 and an ICC_3,1_ of 0.73, respectively. This indicates an almost excellent robustness of the connectivity estimates of the cluster identified to relate to skill learning ability.

#### Structural analysis.

The structural analysis did not provide any evidence for an association between the mean gray matter volume of the 69 subnetwork nodes and skill learning ability (*t*_(56)_ = −0.33, *p* = 0.74). Also, we detected no significant correlations between mean gray matter volume of the 69 subnetwork nodes and the mean functional connectivity estimates of the 91 links of the NBS subnetwork (*t*_(56)_ = −0.10, *p* = 0.92) or any of the four whole-brain graph features identified to relate to skill learning ability (all |*t*_(56)_| < 1.28, *p* > 0.21). This makes the influence of structural confounds on skill learning ability and its association with the identified NBS subnetwork unlikely.

#### Effects of NMDA receptor challenge.

**Figure 4.**
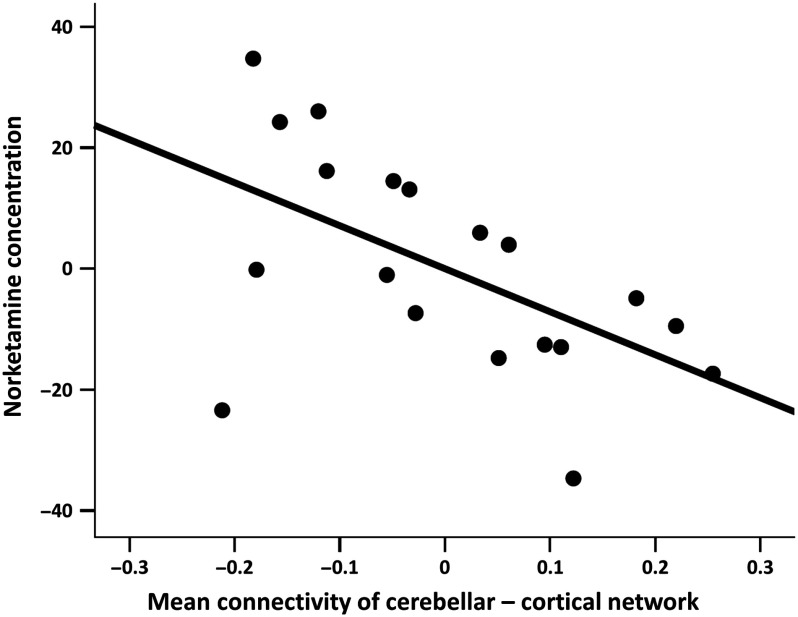
Partial correlation plot of negative correlation between blood Norketamine concentration and the mean connectivity of the NBS-derived cerebellar-cortical network in the ketamine condition (*p* = 0.032; adjusted for covariates and constant), controlled for age, sex, BMI, and FD.

We detected no significant behavioral differences for skill increase (block 4 to block 1, *F*_(1,17)_ = 0.33, *p* = 0.86), task duration (*F*_(1,17)_ = 1.82, *p* = 0.20) or error rate (*F*_(1,17)_ = 0.48, *p* = 0.50) between the placebo and ketamine conditions (drug order was included as covariate of noninterest). In comparison to placebo, application of ketamine did not result in significant differences in global network measures (all *F*_(1,12)_ < 0.83, *p* > 0.38) or whole-brain mean connectivity (*F*_(1,12)_ = 2.04, *p* = 0.18), but significantly decreased the mean functional connectivity of the learning-associated cerebellar-cortical network (*F*_(1,12)_ = 6.38, *p* = 0.027). In addition, the mean connectivity of the cerebellar-cortical network was significantly negatively correlated with individual Norketamine concentrations (46.1 ± 21.6 ng/ml, *t*_(13)_ = −2.40, *r* = −0.55, *p* = 0.032, Figure 4) in the ketamine condition (age, sex, FD, and BMI were controlled as covariates of noninterest). Average FD (*p* = 0.424) and the time interval between drug infusion and resting-state scan (*p* = 0.219) were not significantly different between the ketamine and placebo conditions. A trend toward a main effect of drug order was found (*p* = 0.06). The results indicate that the functional connectivity of the NBS-derived cerebellar-cortical network is affected by ketamine and negatively associated to the concentration of the major active metabolite (i.e., Norketamine).

#### Controlling for individual mean functional connectivity differences.

Using the mean functional connectivity as an additional covariate of noninterests, we replicated the findings that skill increase is significantly positively associated with small-worldness (*p* = 0.008) and global efficiency (*p* = 0.005) and negatively associated with characteristic path length (*p* = 0.002) and transitivity (*p* = 0.006). Similarly, we replicated our NBS results by using the mean correlation as an additional covariate of noninterest. Specifically, we found both statistically equivalent (same uncorrected initial threshold, different number of links in network; FWE corrected *p* < 0.001, 220 links, ICC > 0.75) and density-equivalent (similar number of links in network, but more strict initial threshold *p* < 0.0001; FWE corrected *p* < 0.001, 87 links, ICC > 0.72) cerebellar-cortical subnetworks that were significantly associated with short-term motor learning ability and were modulated by ketamine (*F*_(1,11)_ = 6.97, *p* = 0.023 for the subnetwork with 220 links; *F*_(1,11)_ = 4.93, *p* = 0.048 for the subnetwork with 87 links). Age, sex, order, BMI, mean FD, and mean connectivity were controlled for as covariates of noninterests. Moreover, a marginal significant negative correlation was found between the mean connection of the statistically equivalent subnetwork and the Norketamine concentration (*r* = −0.51, *p* = 0.060), whereas the mean connection of the density-equivalent subnetwork was significantly correlated with the Norketamine concentration (*r* = −0.56, *p* = 0.037).

#### Scrubbing.

Using a more stringent motion correction approach, we could replicate our previous findings that motor learning is positively correlated with small-worldness (*r* = 0.39, *p* = 0.003) and global efficiency (*r* = 0.41, *p* = 0.002) and negatively correlated with transitivity (*r* = −0.39, *p* = 0.003; Table S2, Zang et al., 2018). However, motor learning was no longer correlated with characteristic path length (*r* = −0.24, *p* = 0.074; Table S2, Zang et al., 2018).

In addition, we could replicate our finding of a cerebellar-cortical subnetwork that was correlated with learning rate. In detail, 81 connections linking bilateral cerebellum to visual, sensorimotor, parietal, and frontal areas were positively correlated with skill increase (uncorrected initial *p* < 5 × 10^−4^, FWE corrected *p* = 0.036) while controlling age and sex as covariates of noninterest (FD was no longer controlled as covariate of noninterest since we used FD for scrubbing). This subnetwork also had a fair to good reliability (ICC > 0.73). We then extracted the mean connectivity of the 81 connections and found a significant main effect of drug (*F*_(1,13)_ = 5.10, *p* = 0.042) controlling for age, sex, order, and BMI as covariates of noninterest. The mean connectivity of the 81 connections was also significantly negatively correlated with Norketamine concentrations (*r* = −0.55, *p* = 0.03) when age, sex, and BMI were controlled as covariates of noninterest.

#### Influence of initial threshold definition.

To further explore the robustness of our NBS finding, we repeated our skill learning association analysis by using two different initial thresholds for cluster definition, that is, *p* < 0.001 (less strict) and *p* < 0.0001 (more strict). Notably, a less strict initial *p* value should result in a larger but more unspecific network, whereas a stricter initial *p* value should provide a more specific, but smaller network. As expected, using an initial threshold of *p* < 0.0001, we found a similar but smaller network of 38 links including the left cerebellum and cortical areas that were significantly correlated with skill learning (FWE *p*_*corr*_ = 0.012). Moreover, the *p* < 0.001 initial threshold resulted in a larger, cerebellar-cortical network consisting of 134 links that were significantly correlated with skill learning (FWE *p*_*corr*_ = 0.048). We conclude from these observations that the reported association of the cerebellar-cortical network with skill learning is observed across a range of initial *t* threshold definitions for NBS.

#### Influence of parcellation choice.

To further probe our AAL-based findings for potential effects of parcellation choice, we repeated our analysis with a recently published functional parcellation atlas including 268 nodes (Rosenberg et al., 2016). Notably, the choice of this particular functional atlas was motivated by the fact that it contains a comparable number of node definitions and covers the cerebellum in adequate detail, which is an important prerequisite given that we employed a motor learning paradigm challenging subcortical and cerebellar structures. All other data processing and analysis procedures were kept identical to our initial AAL-based analysis. Similar to our AAL-based analysis, we detected a significant positive correlation of skill increase with small-worldness (*r* = 0.28, *p* = 0.031) and global efficiency (*r* = 0.36, *p* = 0.005) and a significant negative correlation of skill increase with transitivity (*r* = −0.38, *p* = 0.002). In addition, the NBS analysis with the Rosenberg atlas resulted in a very similar but interestingly less reliable (ICC_2,1_ = 0.63, ICC_3,1_ = 0.64) cerebellar-cortical network with 69 links that showed a significant positive association with skill learning (FWE corrected *p* = 0.044, Figure S1, Zang et al., 2018). Moreover, comparable to our AAL-based findings, the mean connectivity of this network was significantly negatively correlated with Norketamine concentrations (*r* = −0.62, *p* = 0.014). We conclude from these observations that (a) our AAL-based NBS findings do not relate to the choice of this particular parcellation scheme, and (b) that the choice of a functional parcellation atlas does not necessarily improve the reliability of the examined connectivity estimates.

## DISCUSSION

In the current resting-state fMRI study, we found several global brain network features to be significantly associated with individual motor learning ability. Furthermore, using a well-established motor learning task (Reis et al., 2009), we identified a cerebellar-cortical functional subnetwork that was (a) significantly associated with short-term learning ability and (b) significantly modulated by NMDA receptor antagonism. We discuss our findings in more detail in the following paragraphs.

First, we demonstrate that short-term motor learning ability is associated with several global network features that characterize a network’s capability to process information efficiently (Bullmore & Sporns, 2009; Rubinov & Sporns, 2010). Specifically, global efficiency and small-worldness were positively correlated with motor learning ability, whereas transitivity and characteristic path length were negatively correlated. Although these learning-associated global network features are highly correlated with each other, they converge on the idea that higher network integration may favor better short-term motor learning ability. This notion is in line with previous studies demonstrating that higher network integration is beneficial for a range of brain functions, including intelligence (van den Heuvel, Stam, Kahn, & Hulshoff Pol, 2009) and working memory (Alavash, Doebler, Holling, Thiel, & Giessing, 2015). Importantly, whereas previous studies have suggested that motor learning changes resting-state connectivity patterns in terms of local network measures (Sami et al., 2014; Zhang et al., 2012), global resting-state network characteristics of the brain have been shown to be relatively stable (Braun et al., 2012; Cao et al., 2014) and untouched by the effects of motor learning (Heitger et al., 2012; Sami & Miall, 2013). Taken together, this may indicate that those global features of brain networks rather reflect the brain’s general capability to master a task independent of training-induced alterations.

Second, we identified a highly plausible cerebellum-centered network with links between cerebellar, visuospatial, sensorimotor, frontal, and temporal regions that were positively associated with an individual’s learning ability. We further provided evidence suggesting that the associated subnetwork is relatively reliable and robust against a variety of potential influencing factors including local gray matter volume, age, sex, head motion, individual mean functional connectivity differences, and the choice of the initial cluster-forming significance threshold and parcellation scheme, respectively.

Notably, the identified subnetwork is highly plausible since it connects several key areas involved in the early phase of visuomotor learning, including M1, SMA, premotor cortex, V5, parietal cortex, and cerebellum (Bassett et al., 2011; Doyon et al., 2002; Hikosaka et al., 2002; Zhang et al., 2012). Among these regions, M1, SMA, premotor, and visual cortex in particular have been related to the computational integration of spatial motor demands (Hikosaka et al., 2002) and the handling of on-line visual feedback (Dong et al., 2012) during the acquisition of complex motor skills. Both functions are crucially important in the early learning phase of our complex motor learning paradigm which requires constant visually guided feedback control and real-time adjustments of executed motor programs. In addition, several parietal regions participated in the cerebellum-centered network, an observation that is in line with the suggested role of these regions in motor imagery learning (Zhang et al., 2012), a key element for planning the upcoming movements’ kinetic parameters (Kuang, Morel, & Gail, 2016). Moreover, the involvement of bilateral DLPFC is consistent with previous motor learning studies (Bassett, Yang, Wymbs, & Grafton, 2015; Heitger et al., 2012) and may plausibly relate to the high level of visual attention demands (Barbey et al., 2013) and complex sequential memory input in motor learning tasks (Toni and Passingham, 1999), especially during the early learning phase (Bassett et al., 2015). Furthermore, the central role of the cerebellum in our identified subcircuit is in good agreement with prior PET and fMRI studies. These studies demonstrated a crucial role of the cerebellum as an error detector and parameter modifier of motor reference plans in early learning phases (Doyon et al., 2002; Penhune & Steele, 2012). This has been evidenced, for example, by severe impairments in certain aspects of motor learning (e.g., reaction time) due to lack of behavioral adjustment in the face of errors in patients with cerebellar lesions (Laforce & Doyon, 2001; Smith & Shadmehr, 2005). Although the observed association between connectivity of the cerebellum-centered subnetwork and motor learning ability could be interpreted as a stronger intrinsic capability of the network architecture in superior learners, it could also be argued that the association is a consequence of learning-induced motor memory consolidation (Albert et al., 2009; Sami et al., 2014) since the resting-state scan was acquired posttraining.

Third, consistent with prior system-level ketamine studies in humans (Kraguljac et al., 2016; Niesters et al., 2012), we found that the cerebellum-centered network was significantly modulated by NMDA receptor antagonism and its connectivity was negatively correlated with blood-level Norketamine concentrations. Interestingly, the motor learning performance before the scan itself was not affected (Francois et al., 2016; van Loon et al., 2016) by low-dose ketamine infusion. Similar observations were made in object-recognition and reward-anticipation fMRI studies, in which authors showed significantly altered BOLD responses but no main effect of drug under low-dose (e.g., ≤0.5 mg/kg) ketamine administration during task performance. This might indicate that the administered drug dose was sufficient to alter neural functional interactions in the identified cerebellum-centered subnetwork, but below the dose level at which overt interruptions of motor learning behavior become evident. In addition, the absence of behavioral differences between the drug conditions suggests that the observed connectivity differences are unlikely to be the consequence of drug-induced changes in motor performance. The detected changes in cerebellar-cortical network connectivity suggest a role for NMDA receptor-dependent glutamatergic neurotransmission that may relate to consolidation processes. This interpretation is consistent with previous reports of a strong dependence of memory consolidation processes (Volianskis et al., 2015), BDNF genotype (Gosselin et al., 2016), and plasticity-related protein synthesis in the motor cortex (Luft, Buitrago, Ringer, Dichgans, & Schulz, 2004). Notably, the fact that we found no modulation of global network measures by ketamine further supports our earlier interpretation of these whole-brain efficiency markers as traitlike reflections of the brain’s capability to perform a range of different tasks.

Our study has several limitations worth mentioning. Most importantly, although our finding of learning-related subnetwork connectivity indices is in line with the hypothesis that motor training leads to temporary changes in the functional brain network architecture, the directionality of such an effect cannot be claimed with our cross-sectional data. Even though we acquired resting-state data *after* off-line motor learning, the interpretation of a predisposed suitability of intrinsic brain networks for the challenged motor performance is equally plausible (Mary et al., 2016). Second, although the connectivity within the motor learning–associated subnetwork was significantly decreased under NMDA receptor blockade, the interpretation of impaired motor memory consolidation would ultimately require an affected motor performance in a preceding motor task. However, as we did not reassess the motor performance after scanning, we must defer such a proof to future studies. Third, previous resting-state studies (Albert, Robertson, & Miall, 2009; Barnes, Bullmore, & Suckling, 2009) provided evidence for an impact of motor and cognitive tasks on the functional configuration of resting-state networks in subsequent MRI scans. This implies that in the case of drug-dependent differences in task engagement prior to the scan, variant carry-over effects (instead of or in addition to an NMDA receptor-related neural plasticity mechanism) may have influenced our drug challenge results. Notably, we did not detect significant main effects of drug condition on behavioral markers of training performance, which argues against such an interpretation. We nonetheless cannot fully exclude that other drug-induced differences in task engagement may have existed and have been carried over to the following resting-state scan. Fourth, although ketamine modulated our specific cerebellum-centered subnetwork, ketamine as a noncompetitive NMDA receptor antagonist may also plausibly influence other brain subnetworks.

In conclusion, we demonstrate that global brain network characteristics and specific subnetwork connectivity patterns during resting state are associated with motor learning *before* scanning. We further show that the identified learning-related subnetwork connectivity estimates are unrelated to the gray matter volume of the nodes, reliable, and susceptible to glutamate challenge. We posit that the observed differential modulation of the examined whole-brain graph theoretical versus cerebellar-cortical network features by ketamine may reflect distinct qualities of learning-related brain function, for example, individual predisposition for learning new motor skills (global brain network measures) versus glutamate-dependent processes related to active motor memory consolidation (cerebellar-cortical network connectivity). Taken together, this investigation may offer valuable information on the neural processes related to short-term motor learning in humans and provide a starting point for future studies in a still under-researched area of human neuroscience.

## ACKNOWLEDGMENTS

We thank Ilka Alexi, Carolin Dennewill, Tobias Gradinger, Jascha Thiem, Canan Koc, Oliver Grimm, Leila Haddad, and Mathias Kienow for research assistance.

## FUNDING INFORMATION

This study was supported by the German Federal Ministry of Education and Research (BMBF, grant 01GQ1102 to H.T.).A.M.-L. acknowledges grant support by the European Community’s Seventh Framework Programme under the grant agreement No. 115008 (Project EU-NEWMEDS).

## AUTHOR CONTRIBUTIONS

Heike Tost, Andreas Meyer-Lindenberg, and Janine Reis: Design of the study. Zhenxiang Zang, Heike Tost, Lena Geiger, and Urs Braun: Writing. Zhenxiang Zang, Urs Braun, Axel Schäfer, and Emanuel Schwarz: Data analysis. Zhenxiang Zang, Lena Geiger, Maria Zangl, and Janina I. Schweiger: Data collection. Axel Schäfer, Matthias Ruf, and Janine Reis: Provided of experiment materials. Hengyi Cao, Carolin Moessang, Luanna Dixson, Alexander Moscicki, and Andreas Meyer-Lindenberg: Manuscript revision.

## CONFLICT OF INTEREST

A.M.-L. has received consultant fees and travel expenses from Alexza Pharmaceuticals, AstraZeneca, Bristol-Myers Squibb, Defined Health, Decision Resources, Desitin Arzneimittel, Elsevier, F. Hoffmann-La Roche, Gerson Lehrman Group, Grupo Ferrer, Les Laboratoires Servier, Lilly Deutschland, Lundbeck Foundation, Outcome Sciences, Outcome Europe, PriceSpective; and Roche Pharma and has received speaker’s fees from Abbott, AstraZeneca, BASF, Bristol-Myers Squibb, GlaxoSmithKline, Janssen-Cilag, Lundbeck, Pfizer Pharma, and Servier Deutschland. The other authors report no biomedical financial interests or other potential conflicts of interest.

## TECHNICAL TERMS

*N*-methyl-d-aspartate receptor (NMDA): NMDA receptor is a glutamate receptor (ion channel protein) in the central nervous system that is very important for synaptic plasticity and memory function.
Ketamine: Ketamine is a noncompetitive NMDA receptor antagonist that binds specifically to a subsite of NMDA receptor and blocks ion channels in the central nervous system. It is normally used to induce and maintain general anesthesia before, during, and after surgery.
Sequential visual isometric pinch force task: Subjects are required to pinch their right thumb and index finger against a force transducer to achieve the “target” by looking at the cursor displayed on the monitor.
Skill measure: Skill measure is an index to reflect the performance level by combining speed and accuracy with a nonlinear formula.
Network-based statistics (NBS): NBS is a method to perform group level statistics on correlation matrix by using a general linear model. An arbitrary initial threshold is needed to determine the network cluster, and permutations will be performed on the determined network for addressing multiple comparison correction.
Global network features: In the current study, global network features are defined as small-worldness, global efficiency, characteristic path length, modularity Q, transitivity, assortativity, and mean correlation coefficient.
