# Supplementary material for: Resting-state brain network features associated with short-term skill learning ability in humans and the influence of N-methyl-d-aspartate receptor antagonism
Source: Netw Neurosci. 2018 Oct 1;2(4):464–80. doi: 10.1162/netn_a_00045 (PMC6175691; doi:10.1162/netn_a_00045)
Supplement: Supplementary file 1 [file netn-02-464-s001.pdf]

Zang, Z., Geiger, L.S., Braun, U., Cao, H., Zang, M., Schäfer, A., … & Tost, H. (2018). Supporting Information for "Resting-state brain network features associated with shorterm skill learning ability in humans and the influence of N-mythyl-aspertate receptor antagonism." *Network Neuroscience,* 2(4), 464-480. https://doi.org/10.1162/netn_a_00045

Supplemental Materials

RESTING-STATE BRAIN NETWORK FEATURES ASSOCIATED WITH SHORT-TERM SKILL LEARNING ABILITY IN HUMANS AND THE INFLUENCE OF N-METHYL-D-ASPARTATE RECEPTOR ANTAGONISM

Zang ZX., Geiger L.S., Braun.U,.., Heike Tost, et al.

S-Method

MRI data acquisition parameters

Resting-state fMRI was performed using an echo-planar imaging (EPI) sequence with the following parameters: TR = 1790 ms, TE = 28 ms, 34 axial slices per volume, voxel size = 3 x 3 x 3 mm, 1 mm slice gap, 192 x 192 mm field of view, and 76° flip angle, descending acquisition. For the resting-state experiment, we instructed participants to close their eyes, relax, and not engage in any particular mental activity during the scan (task duration: 5 minutes, 167 whole-brain scans). After each scan, we confirmed with the subjects that they had not fallen asleep in the scanner. In addition, we acquired high-resolution T1-weighted 3-dimensional images with a magnetization-prepared rapid gradient echo sequence (3D-MPRAGE) and the following parameters: TR = 2530 ms, TE = 3.8 ms, TI = 1100 ms, 176 slices, 256 x 256 mm field of view, 7° flip angle, and 1 mm^3 spatial resolution.

Global graph diagnostics

Characteristic path length is the average shortest path length between each pair of nodes in the network, with shorter path lengths indicating faster information flow. *Global efficiency* is the average of the inverse shortest path length and relates to the capacity of the network for information transfer at the global level. *Smallworldness* is the ratio of the normalized

clustering coefficient (segregation) and normalized path length (integration) (Bullmore & Sporns, 2009). In the current study, 100 randomized networks were generated to calculate smallworldness. *Modularity Q* is the degree to which the network can be partitioned into non-overlapping modules, with higher values indicating a more developed network community structure with more densely connected local nodes. *Assortativity* reflects how often nodes of a similar degree are connected with each other. And *transitivity* reflects the global clustering coefficient of nodes (Rubinov & Sporns, 2010). The underlying methods and interpretation of these metrics are discussed in more detail elsewhere (Bassett & Bullmore, 2006; Bullmore & Sporns, 2009; Rubinov & Sporns, 2010).

Test-retest reliability data

In this study, 26 healthy volunteers (mean age: 24.4 ± 2.8 years, 15 females) underwent a 5 minute resting-state scan twice within two consecutive weeks (mean interval: 14.6 ± 2.1 days). All 26 volunteers were naive to the current motor learning task. The processing of data, definition of nodes, and construction of connectivity matrices were consistent with the current work. We extracted the mean connectivity estimates from the links identified in the current NBS analysis (see results section) and calculated intra-class correlation coefficients (ICCs) as indices of robustness.

Structural correlates

Briefly, the processing of images included tissue classification, normalization to MNI space with a diffeomorphic image registration algorithm, correction for image intensity non-uniformity, a thorough cleaning up of gray matter partitions, application of a hidden Markov random field model, transformation of GM density values into volume equivalents, and smoothing with an 8mm FWHM Gaussian kernel.

S-Results

Table S1

Node	Coordinates	T value	P value	Node	Coordinates	T value	P value
**CEREBELLUM.7B.L (-43, -54, -48)**							
**Frontal.Sup.R[2]**	**32, -11, 67**	**4.35**	**2.92×10^{-5}**	SMA.R[4]	5, -19, 58	3.83	1.64×10^{-4}
**Frontal.Sup.L[2]**	**-27, -8, 60**	**4.55**	**1.52×10^{-5}**	SMA.L[4]	-5, -21, 52	3.58	3.59×10^{-4}
	**-16, 1, 71**	**4.20**	**4.88×10^{-5}**	**PreCentral.R[3]**	**59, 3, 26**	**4.55**	**1.52×10^{-5}**
	**-20, -5, 53**	**4.04**	**8.28×10^{-5}**		**21, -15, 71**	**4.11**	**6.74×10^{-5}**
**Frontal.Mid.R[2]**	**49, -2, 53**	**4.49**	**1.83×10^{-5}**		40, -7, 47	3.88	1.43×10^{-4}
	30, -2, 56	3.81	1.74×10^{-4}	**PreCentral.L[3]**	**-24, -20, 74**	**4.59**	**1.33×10^{-5}**
Frontal.Inf.Tri.R[7]	50, 16, 25	3.71	2.41×10^{-4}		**-43, -1, 56**	**4.38**	**2.70×10^{-5}**
Cuneus.R	2, -85, 31	3.53	4.23×10^{-4}		**-35, -24, 67**	**4.03**	**8.57×10^{-5}**
**Rolandic.Oper.L[5]**	**-59, 2, 11**	**4.18**	**5.33×10^{-5}**	**PostCentral.R[3]**	**49, -31, 56**	**5.13**	**1.98×10^{-6}**
**Parietal.Sup.R[6]**	**-16, -59, 71**	**4.76**	**7.18×10^{-6}**		**38, -28, 46**	**4.08**	**7.41×10^{-5}**
	**13, -72, 54**	**4.19**	**5.09×10^{-5}**		**29, -40, 70**	**4.05**	**8.1×10^{-5}**
	**24, -63, 52**	**4.17**	**5.49×10^{-5}**		**45, -20, 60**	**4.04**	**8.28×10^{-5}**
	**15, -47, 66**	**4.09**	**7.20×10^{-5}**		63, -8, 25	3.66	2.81×10^{-4}
**Parietal.Sup.L[6]**	**-30, -52, 62**	**4.64**	**1.10×10^{-5}**	**PostCentral.L[3]**	**-45, -23, 47**	**4.29**	**3.59×10^{-5}**
	-17, -73, 51	3.51	4.58×10^{-4}		**-56, -2, 38**	**4.20**	**4.96×10^{-5}**
**Parietal.Inf.L**	**-49, -36, 48**	**4.28**	**3.72×10^{-5}**		**-34, -32, 55**	**4.01**	**9.22×10^{-5}**
Paracentral.Lobule.L	-9, -30, 66	3.67	2.75×10^{-4}	**Occipital.Sup.R[1]**	**25, -80, 41**	**4.62**	**1.16×10^{-5}**
**Precuneus.L**	**-13, -54, 67**	**4.75**	**7.36×10^{-6}**	**Occipital.Mid.R**	**34, -81, 31**	**4.55**	**1.49×10^{-5}**
	**-11, -63, 63**	**4.36**	**2.92×10^{-5}**	**Occipital.Mid.L**	**-29, -83, 25**	**4.08**	**7.32×10^{-5}**
**Calcarine.R**	**13, -71, 19**	**4.00**	**9.41×10^{-5}**		-44, -74, 5	3.88	1.37×10^{-4}
	23, -66, 9	3.77	1.98×10^{-4}	**Temporal.Mid.R**	**46, -71, 13**	**4.18**	**5.28×10^{-5}**
	12, -81, 14	3.49	4.81×10^{-4}		51, -62, 1	3.93	1.19×10^{-4}
Calcarine.L	-4, -91, -7	3.95	1.11×10^{-4}	**Lingual.L**	**-8, -80, -10**	**4.75**	**7.50×10^{-6}**
	-7, -95, 9	3.61	3.29×10^{-4}	**SupraMarginal.R**	**58, -23, 41**	**4.13**	**6.23×10^{-5}**
**CEREBELLUM.8.L (-26, -64, -48)**							

Frontal.Sup.R[2]	32, -11, 67	3.84	1.59×10^{-4}	**PreCentral.R[3]**	**59, 3, 26**	**4.34**	**3.05×10^{-5}**
Frontal.Sup.L[2]	-27, -8, 60	3.94	1.16×10^{-4}		40, -7, 47	3.56	3.81×10^{-4}
	-20, -5, 53	3.86	1.50×10^{-4}	**PreCentral.L[3]**	**-43, -1, 56**	**4.16**	**5.69×10^{-5}**
	-16, 1, 71	3.50	4.65×10^{-4}	PostCentral.L[3]	-56, -2, 38	3.81	1.77×10^{-4}
**Frontal.Mid.R[2]**	**49, -2, 53**	**4.40**	**2.50×10^{-5}**		-34, -32, 55	3.62	3.17×10^{-4}
Calcarine.R	23, -66, 9	3.67	2.71×10^{-4}	**Lingual.L**	**-8, -80, -10**	**4.11**	**6.74×10^{-5}**
	13, -71, 19	3.53	4.25×10^{-4}		-24, -71, 0	3.54	4.10×10^{-4}
**Temporal.Sup.R**	**50, -33, 10**	**4.09**	**7.07×10^{-5}**	SMA.R[4]	5, -19, 58	3.55	3.98×10^{-4}
Temporal.Mid.R	46, -71, 13	3.59	3.48×10^{-4}	SMA.L[4]	-1, -2, 57	3.52	4.33×10^{-4}
Temporal.Pole.Mid.R	26, 6, -38	3.50	4.72×10^{-4}	Rolandic.Oper.L[5]	-59, 2, 11	3.78	1.94×10^{-4}
**CEREBELLUM.8.R (38, -42, -48)**							
Lingual.L	-8, -80, -10	3.63	3.07×10^{-4}				
**CEREBELLUM.Crus1.R (38, -74, -31)**				**CEREBELLUM.Crus1.L (-39, -76, -33)**			
Temporal.Mid.L	-64, -14, -11	3.95	1.13×10^{-4}	**Temporal.Mid.L**	**-64, -14, -11**	**4.08**	**7.39×10^{-5}**
	-53, -33, -10	3.52	4.38×10^{-4}	Lingual.L	-8, -80, -10	3.84	1.58×10^{-4}
				Calcarine.L	-4, -91, -7	3.52	4.31×10^{-4}
**CEREBELLUM.Crus1.R (37, -49, -37)**							
Lingual.L	-8, -80, -10	3.76	2.06×10^{-4}	**CEREBELLUM.Crus1.L (-50, -55, -38)**			
Calcarine.R	23, -66, 9	3.53	4.29×10^{-4}	Lingual.L	-8, -80, -10	3.53	4.28×10^{-4}
**CEREBELLUM.Crus2.R (42,-65,-48)**							
**Frontal.Inf.Tri.L[7]**	**-53, 26, 20**	**4.18**	**5.34×10^{-5}**	**Temporal.Inf.L**	**-52, -13, -28**	**4.93**	**3.95×10^{-6}**
PreCentral.L[3]	-43, -1, 56	3.76	2.06×10^{-4}		-38, 5, -36	3.90	1.31×10^{-4}
Parietal.Sup.L[6]	-30, -52, 62	3.92	1.25×10^{-4}	Fusiform.L	-27, 1, -39	3.58	3.6×10^{-4}
Occipital.Sup.R[1]	25, -80, 41	3.50	4.66×10^{-4}				
**Temporal.Mid.L**	**-53, -33, -10**	**4.65**	**1.05×10^{-5}**	**CEREBELLUM.Crus2.L (-24, -75, -72)**			
	**-56, 1, -27**	**4.06**	**7.92×10^{-5}**	Frontal.Sup.L[2]	-20, -5, 53	3.53	4.24×10^{-4}
	**-64, -41, -13**	**4.04**	**8.49×10^{-5}**	Frontal.Mid.R[2]	49, -2, 53	3.84	1.58×10^{-4}
	**64, -14, -11**	**4.03**	**8.91×10^{-5}**	**Frontal.Inf.Tri.R[7]**	**50, 16, 25**	**4.46**	**2.03×10^{-5}**

Table S1. Bolded letters indicate the links with the strongest ($P < 1 \times 10^{-4}$) individual associations with skill learning ability. Map coordinates refer to the center of gravity of the nodes and the standard space defined by the Montreal Neurological Institute, MNI. The corresponding label of the AAL atlas was assigned to each node for orientation. Numbers in square brackets indicate nodes mapping in proximity to: [1] visual motion-sensitive areas V5,

[2] dorsal premotor cortex, [3] primary sensory-motor cortex, [4] supplementary motor area, [5] ventral premotor cortex, [6] intraparietal sulcus, [7] dorsolateral prefrontal cortex.

Table S2

	Original results		Mean connectivity corrected		Scrubbing	
Network features	T values	P_raw	T values	P_raw	T values	P_raw
Smallworldness	2.73	0.008	2.74	0.008	3.13	0.003
Global efficiency	2.90	0.005	2.92	0.005	3.28	0.002
Path length	-3.33	0.002	-3.20	0.002	-1.82	0.074
Modularity Q	1.55	0.126	1.29	0.203	2.32	0.024
Assortativity	-1.82	0.075	1.89	0.064	-2.21	0.031
Transitivity	-2.92	0.005	-2.84	0.006	-3.13	0.003
Mean connection	-0.84	0.406	/	/	-0.73	0.443

Table S2. List of T values and P values on the association between motor learning ability and graph properties for original results, mean correlation corrected results and scrubbing results.

Figure S1

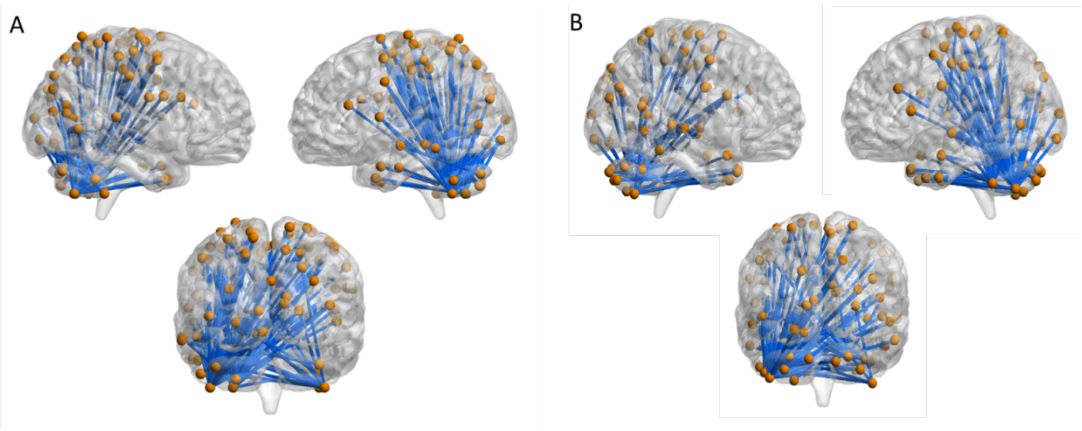

Figure S1. Panel A: Learning related cerebellum – cortical network identified based on AAL parcellation. In summary, 91 links were significantly correlated with skill learning (FWE p = 0.037) Panel B: Replication of cerebellum – cortical network using functional parcellation. In summary, 69 links were significantly correlated with skill learning (FWE p = 0.044). Displayed nodes represent the coordinates of the center of gravity of the sub-regions.

References

Bassett, D. S., & Bullmore, E. (2006). Small-world brain networks. *Neuroscientist, 12*(6), 512-523. doi:10.1177/1073858406293182

Bullmore, E., & Sporns, O. (2009). Complex brain networks: graph theoretical analysis of structural and functional systems. *Nat Rev Neurosci, 10*(3), 186-198. doi:10.1038/nrn2575

Rubinov, M., & Sporns, O. (2010). Complex network measures of brain connectivity: uses and interpretations. *Neuroimage, 52*(3), 1059-1069. doi:10.1016/j.neuroimage.2009.10.003
